# Author Correction: Intravital imaging of islet Ca^2+^ dynamics reveals enhanced β cell connectivity after bariatric surgery in mice

**DOI:** 10.1038/s41467-021-25887-8

**Published:** 2021-09-20

**Authors:** Elina Akalestou, Kinga Suba, Livia Lopez-Noriega, Eleni Georgiadou, Pauline Chabosseau, Alasdair Gallie, Asger Wretlind, Cristina Legido-Quigley, Isabelle Leclerc, Victoria Salem, Guy A. Rutter

**Affiliations:** 1 Section of Cell Biology and Functional Genomics, Imperial College London, Hammersmith Hospital Campus, London, UK; 2 Central Biological Services (CBS) Hammersmith Hospital Campus, London, UK; 3 Systems Medicine, Steno Diabetes Center, Gentofte, Copenhagen, Denmark; 4 Section of Investigative Medicine, Division of Diabetes, Endocrinology and Metabolism, Department of Metabolism, Digestion and Reproduction, Imperial College London, Hammersmith Hospital Campus, London, UK; 5 Lee Kong Chian Imperial Medical School, Nanyang Technological University, Singapore, Singapore; 6 Centre de Recherches du CHUM, University of Montreal, Montreal, QC Canada

**Keywords:** Biological techniques, Physiology, Diabetes

Correction to: *Nature Communications* 10.1038/s41467-021-25423-8, published online 27 August 2021.

The original version of this Article contained an error in the name of the author Cristina Legido-Quigley, which was incorrectly given as Cristina Quigley. This has now been corrected in both the PDF and HTML versions of the Article.

