# Supplementary material for: Intravital imaging of islet Ca2+ dynamics reveals enhanced β cell connectivity after bariatric surgery in mice
Source: Nat Commun. 2021 Aug 27;12:5165. doi: 10.1038/s41467-021-25423-8 (PMC8397709; doi:10.1038/s41467-021-25423-8)
Supplement: Supplementary file 1 — Supplementary Information [file 41467_2021_25423_MOESM1_ESM.pdf]

Supplementary Information for:

NComms-20-19605.R2

Intravital imaging of islet Ca^{2+} dynamics reveals enhanced β cell connectivity after bariatric surgery in mice

Elina Akalestou PhD[1], Kinga Suba PhD[1], Livia Lopez-Noriega PhD[1], Eleni Georgiadou PhD[1], Pauline Chabosseau PhD[1], Alasdair Gallie BVM BVS[2], Asger Wretlind PhD[3], Cristina Quigley PhD[3], Isabelle Leclerc MD PhD[1], Victoria Salem MD PhD[1,4]*, and Guy A. Rutter PhD[1,5,6]*

[1] Section of Cell Biology and Functional Genomics, Imperial College London, Hammersmith Hospital Campus, Du Cane Road, W12 0NN London, United Kingdom, [2] Central Biological Services (CBS) Hammersmith Hospital Campus, Du Cane Road, W12 0NN London, United Kingdom [3] Systems Medicin, Steno Diabetes Center Niels Steensens Vej 2, 2820 Gentofte Copenhagen, Denmark, [4] Section of Investigative Medicine, Division of Diabetes, Endocrinology and Metabolism, Department of Metabolism, Digestion and Reproduction, Imperial College London, Hammersmith Hospital Campus, Du Cane Road, W12 0NN London, United Kingdom, [5] Lee Kong Chian School of Medicine, Nanyang Technological University, 11 Mandalay Road 308232, Singapore, [6] CR-CHUM, University of Montreal, 900, rue Saint-Denis, H2X 0A9, QC, Canada

Corresponding Authors:
Guy A. Rutter, Room 327, ICTEM Building, Imperial College London, Hammersmith Hospital Campus, Du Cane Road, W12 0NN London, United Kingdom, phone number: +44 (0)20 7594 3340 g.rutter@imperial.ac.uk
Victoria Salem, 6th Floor Commonwealth Building, Imperial College London, Hammersmith Hospital Campus, Du Cane Road, W12 0NN London, United Kingdom, v.salem@imperial.ac.uk

This PDF file includes:

Supplementary text
Figures S1 to S5
Legends for Movies S1A to S1F

Supplementary Figure 1:

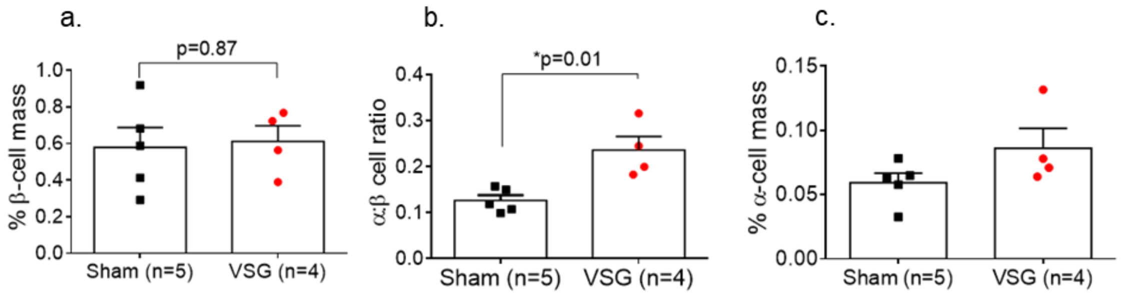

Percentage of pancreatic area occupied by and ratio of α to β cells. (a) Percentage of pancreatic area occupied by β cells measured by immunofluorescent staining of pancreatic islets using anti-insulin antibody against total pancreatic area. (b) Ratio of α to β cells in pancreatic islets of VSG (n=4) and Sham (n=5) mice measured by immunofluorescent staining of pancreatic islets using anti-glucagon (red) and anti-insulin (green) antibody. (c) Percentage of pancreatic area occupied by α cells measured by immunofluorescent staining of pancreatic islets using anti-glucagon antibody against total pancreatic area. All pancreatic biopsies were obtained 10 weeks following surgery. Each point represents an average of all islets (10-30/ slide) present in three permeabilised pancreatic slices, separated by 600μm. Total pancreatic area is measured in each slide and percentage is calculated accordingly.*P<0.05, by unpaired Student's t-test. Data are expressed as means ± SEM. Source data are provided as a Source Data file.

Supplementary Figure 2:

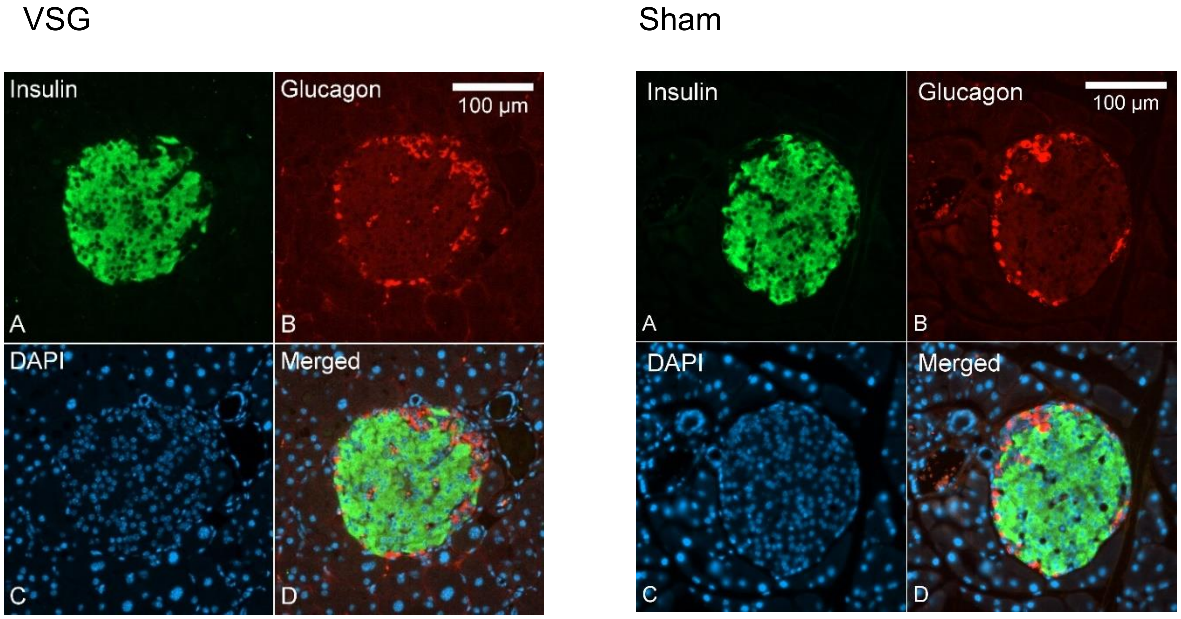

Immunofluorescence staining of pancreatic islets. Anti-glucagon (red) and anti-insulin (green) antibodies where used in VSG (n=4) and Sham (n=5) mice. All pancreatic biopsies were obtained 10 weeks following surgery (age: 32 weeks). For further details please see Results and Methods Sections.

Supplementary Figure 3:

a.

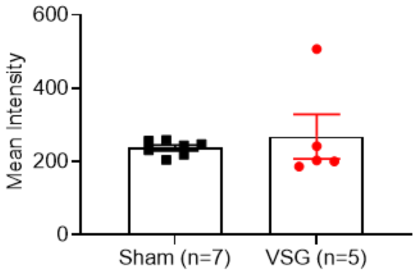

Sham

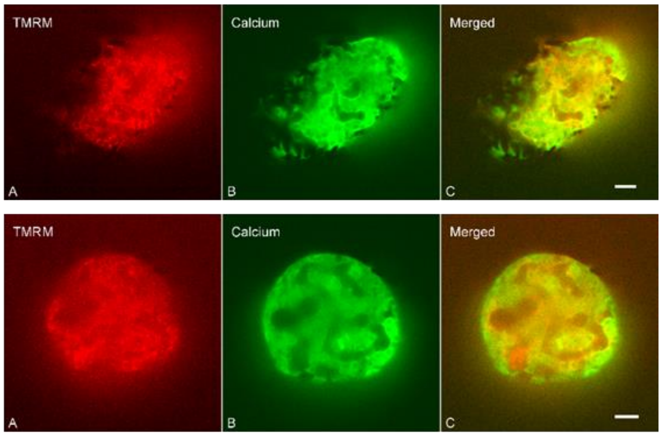

VSG

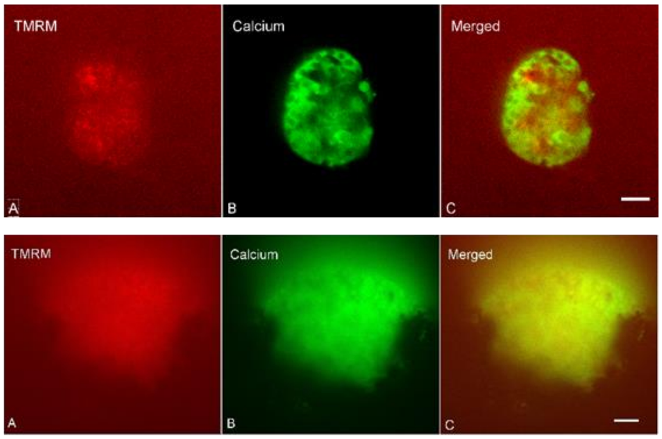

In vivo measurement of mitochondrial membrane potential. Mean Intensity measurements in ACE islets of sham (n=7 islets, 1 mouse) and VSG (n=4 islets, 3 mice) 15 mins following IV injection of TMRM (0.5mg/kg). For further details please see Results and Methods Section. Source data are provided as a Source Data file.

Supplementary Figure 4:

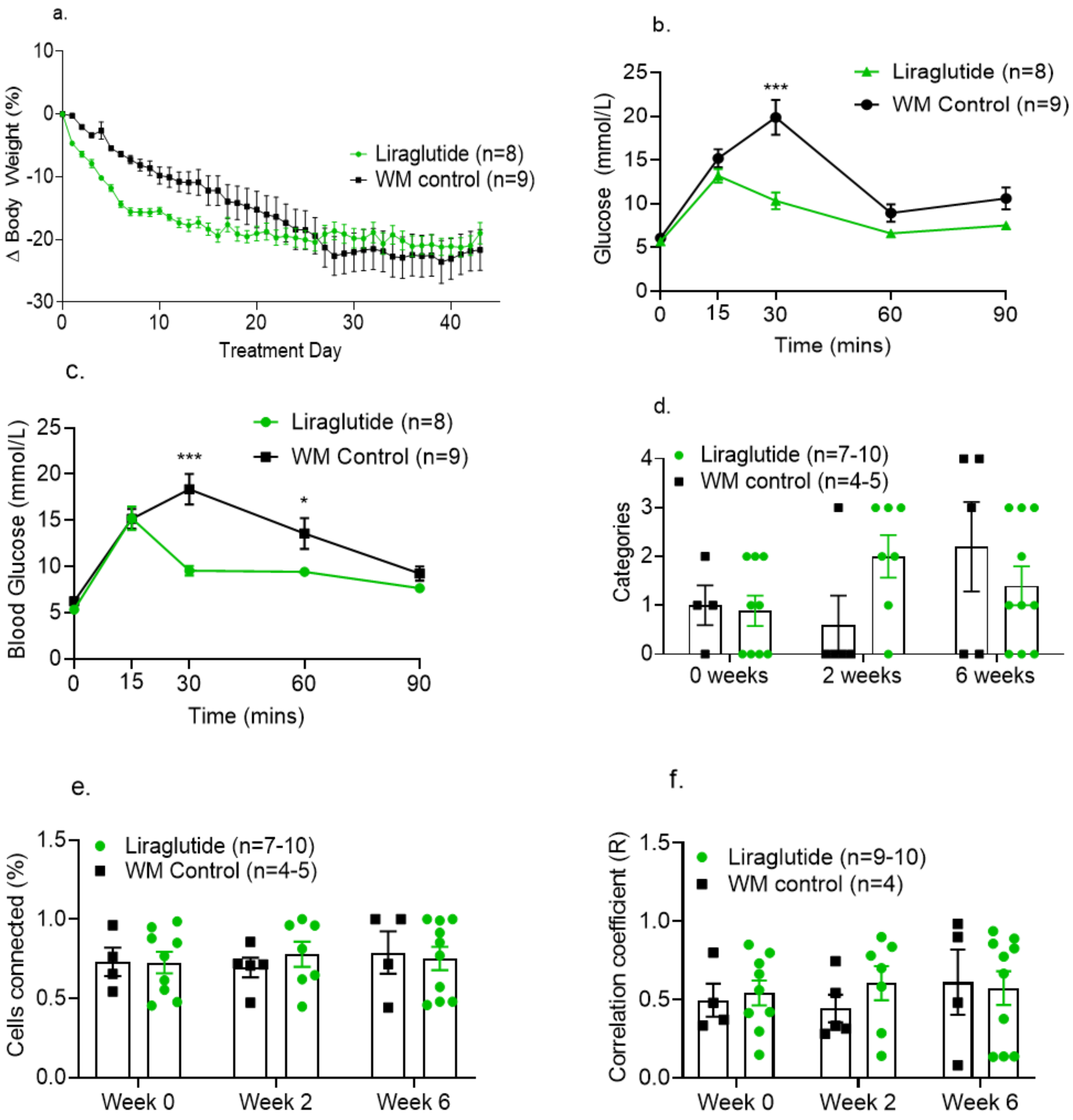

Liraglutide-treated and weight matched group. (a) Body weight monitoring following Liraglutide (n=8 animals) or weight matched control (n=9 (b). Glucose tolerance test. Glucose was administered via intraperitoneal injection (1 g/kg) after mice were fasted overnight and blood glucose levels measured at 0, 15, 30, 60 and 90 min. post injection, after two weeks of treatment and (c) six weeks of treatment (d) Categorisation of Ins1Cre:GCaMPffl/fl islets in Liraglutide-treated (n=6 animals, n=7-10 islets) and weight matched (n=3 animals, n-4-5 islets) mice on weeks 0 and 6. Categories: 0. No activity, 1. Oscillations, 2. Partial Wave, 3. Wave, 4. Super Wave (e) Percentage of significantly connected cell pairs Liraglutide-treated (n=6 animals, n=7-10 islets) and weight matched (n=3 animals, n-4-5 islets) mice (f) Correlation coefficient (R) of Liraglutide-treated (n=6 animals, n=9-10 islets) and weight matched (n=3 animals, n-4 islets) mice. Data are means ± SEM and *p<0.05 by Student t-test. Data are expressed as means ± SEM. Source data are provided as a Source Data file.

Supplementary Figure 5:

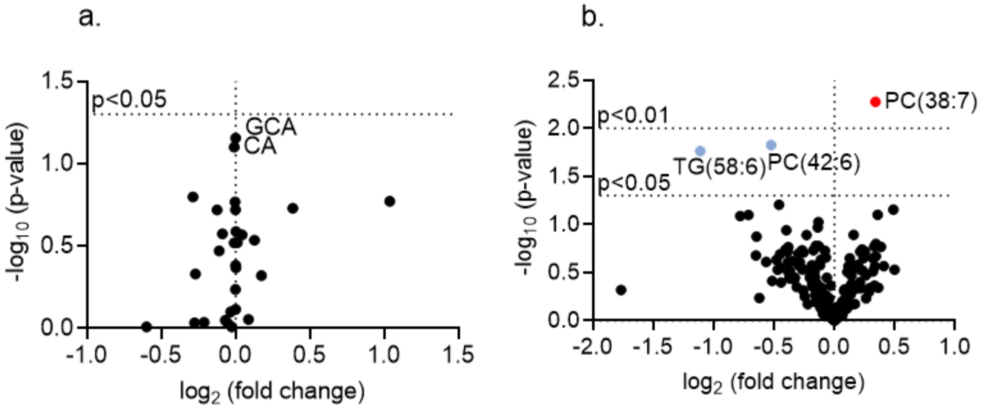

Metabolomics and Lipidomics analysis. (a) Metabolomics and (b) Lipidomics in plasma 12 weeks following VSG (n=3) or sham (n=3) procedures and post 3-day Exendin9 wash out period. Source data are provided as a Source Data file.
