# Supplementary material for: Intravital imaging of islet Ca2+ dynamics reveals enhanced β cell connectivity after bariatric surgery in mice
Source: Nat Commun. 2021 Aug 27;12:5165. doi: 10.1038/s41467-021-25423-8 (PMC8397709; doi:10.1038/s41467-021-25423-8)
Supplement: Supplementary file 3 — Description of Additional Supplementary Files [file 41467_2021_25423_MOESM3_ESM.docx]

**Description of Additional Supplementary Files**

File Name: Supplementary Mov1A

Description: Ins1Cre:GCaMPffl/fl islet implanted in a sham animal, imaged using a spinning disk confocal microscope at week 0 (full wave)

File Name: Supplementary Mov1. B

Description: As A, at week 4 (full wave)

File Name: Supplementary Mov1. C.

Description: As A, at week 12 (inactive).

File Name: Supplementary Mov1. D.

Description: Ins1Cre:GCaMPffl/fl islet implanted in VSG-treated animal, imaged at week 0 (partial wave)

File Name: Supplementary Mov1. E.

Description: As D, week 4 (full wave)

File Name: Supplementary Mov1. F.

Description: As, D, at week 12 (super wave).
